# Concerns from bench and insights from bedside: the puzzle of Roxadustat in cancer patients with chemotherapy-induced anemia

**DOI:** 10.3389/fphar.2025.1703424

**Published:** 2025-11-12

**Authors:** Lin Chen, Shigen Liao, Dan Jing, Linxiu Mao, Jing Tan

**Affiliations:** 1 School of Clinical Medicine, North Sichuan Medical College, Nanchong, Sichuan, China; 2 Department of Hematology, The Third People’s Hospital of Chengdu, Chengdu, Sichuan, China

**Keywords:** roxadustat, HIF-1α, chemotherapy-induced anemia, solid tumors, metabolic reprogramming

## Abstract

Roxadustat, a hypoxia-inducible factor prolyl hydroxylase inhibitor (HIF-PHI), is indicated for the treatment of renal anemia. Its therapeutic mechanism involves stabilizing hypoxia-inducible factor-α (HIF-α), thereby stimulating erythropoietin production and regulating iron metabolism. Recent clinical studies have demonstrated that Roxadustat exhibits efficacy comparable to that of erythropoiesis-stimulating agents (ESAs) in the management of chemotherapy-induced anemia (CIA). However, preclinical studies demonstrate HIF-1α activation promotes tumor progression via multiple pathways (metabolic reprogramming, angiogenesis, metastasis, apoptosis resistance, immune evasion, chemoresistance). Current evidence shows no increased malignancy risk with Roxadustat in renal patients. However, while tumor progression events were reported as treatment-emergent serious adverse events (TESAEs) in CIA studies, clinical data linking Roxadustat to tumor progression remain limited. Furthermore, the observation periods in these studies have been short. A causal relationship remains unestablished due to the insufficient duration of observation required to adequately assess potential HIF-driven oncogenic risks. Consequently, Roxadustat poses a clinical dilemma: its efficacy in CIA offers a promising ESAs alternative, but its HIF-driven oncogenic potential necessitates long-term safety assessment in cancer patients. Future studies must prioritize longitudinal monitoring to define the benefit-risk profile.

## Introduction

CIA is a prevalent complication among cancer patients undergoing chemotherapy, significantly affecting their quality of life and treatment outcomes. The incidence and severity of CIA vary widely depending on the type of cancer, chemotherapy regimen, and individual patient factors (Bryer and Henry, 2018).

Traditional management with erythropoiesis-stimulating agents (ESAs) has improved transfusion dependence, but concerns over venous thromboembolism and tumor progression have limited their use in cancer patients (Abdel-Razeq and Hashem, 2020). These safety concerns have stimulated the pursuit of alternatives to ESAs. Among emerging options, HIF-PHIs represent a novel approach (Lu et al., 2025). These small molecules act by stabilizing HIF-1α through inhibition of prolyl hydroxylase domain (PHD) enzymes, thereby preventing its oxygen-dependent degradation via the von Hippel–Lindau (VHL) ubiquitin-proteasome pathway. Under normoxia, PHDs hydroxylate HIF-1α (at Pro402 and Pro564), allowing VHL to recognize and ubiquitinate HIF-1α for proteasomal degradation. When PHDs are inhibited—either by hypoxia or by HIF-PHIs—HIF-1α accumulates, translocates to the nucleus, and activates downstream target genes, including EPO (Ivan et al., 2001). Currently, multiple HIF-PHIs have entered clinical application or research and development stages globally, such as Roxadustat, Vadadustat, Daprodustat, Belzutifan, Desidustat, Molidustat Sodium and Enarodusta, primarily focusing on renal anemia and cancer treatment (Li et al., 2025). Among these, Roxadustat, as the world’s first HIF-PHI to be approved for marketing, holds a unique status. It demonstrates the feasibility of treating renal anemia with oral medications, breaking the traditional reliance on injectable recombinant human erythropoietin.

Pharmacologically, Roxadustat belongs to this innovative class of drugs that function by stabilizing HIF, a master transcription factor orchestrating the body’s adaptation to hypoxia (Provenzano et al., 2016). Roxadustat regulates the HIF pathway, increasing the expression of endogenous erythropoietin (EPO), thereby stimulating erythropoiesis and improving iron metabolism (Pergola et al., 2022). Roxadustat has been shown to significantly increase hemoglobin levels in patients with chronic kidney disease and reduce the need for intravenous iron supplementation (Ganz et al., 2023). Recently, results from a phase III clinical study evaluating Roxadustat for the treatment of CIA in patients with non-myeloid malignancies were released. The findings demonstrated that oral Roxadustat is non-inferior to ESAs in managing anemia in patients with non-myeloid malignancies receiving multi-cycle myelosuppressive chemotherapy (Lu et al., 2025).

However, given the compelling evidence of a strong correlation between elevated levels of HIF-1 and tumor metastasis, angiogenesis, poor patient prognosis, and tumor resistance, concerns regarding the potential off-target effects of HIF-PHD inhibitors have been raised (Tan et al., 2025).

Building upon the regulatory framework of HIF-1α under normoxia and hypoxia, it becomes essential to understand how this master transcription factor reprograms tumor biology. The following section systematically summarizes the key molecular mechanisms through which HIF-1α contributes to cancer progression.

## The pro-tumor molecular mechanism of HIF-1α

HIF-1 is a heterodimeric transcription factor composed of α and β subunits, both of which are basic helix-loop-helix proteins. The α subunit, known as HIF-1α, exhibits markedly increased expression under hypoxic conditions, while its levels remain low in most cells under normoxic conditions. HIF-1α is crucial for cellular adaptation to the low oxygen levels frequently encountered in the tumor microenvironment. Upon activation in hypoxic settings, HIF-1α initiates a transcriptional program that facilitates invasion, angiogenesis, metabolic reprogramming, and cell survival, collectively contributing to tumor growth and metastasis (Lee et al., 2004).

Genes induced by hypoxia and containing functionally significant HIF-1 binding sites include those encoding erythropoietin, transferrin, vascular endothelial growth factor (VEGF), inducible nitric oxide synthase, heme oxygenase 1, and endothelin 1. Importantly, the protein products of many of these genes are associated with diverse biological functions, including cellular energy metabolism, angiogenesis, and cell survival (Yu et al., 1999).

Research indicates that HIF-1α expression is observed in approximately 83.7% of small cell lung cancer patients, with 60.4% of these individuals exhibiting elevated levels of expression. Furthermore, the mortality risk for patients with high HIF-1α expression is approximately 39.2 times greater (Lin et al., 2017).

## Metabolic reprogramming: energy supply optimization

Metabolic reprogramming constitutes a hallmark of cancer, enabling tumor cells to adapt to challenging microenvironmental conditions, including hypoxia and nutrient deprivation. This adaptive mechanism is modulated by various factors, with HIF-1α serving as a principal mediator in orchestrating these metabolic alterations (Navarro et al., 2022). A defining feature of cancer metabolism is the Warburg effect, distinguished by a preference for glycolysis over oxidative phosphorylation, even in the presence of ample oxygen. This metabolic adaptation enables cancer cells to rapidly produce energy and generate metabolic intermediates essential for proliferation. The increased uptake of glucose and its subsequent conversion to lactate lead to lactate accumulation, even under aerobic conditions. HIF-1α becomes activated under hypoxic conditions and induces the expression of a variety of genes involved in metabolic reprogramming. Through its regulatory role, HIF-1α facilitates the adaptation of cancer cells to environments characterized by low oxygen and nutrient availability. The upregulation of glycolytic enzymes facilitates the glycolytic pathway, while the induction of glucose transporters, such as GLUT1, augments glucose uptake (Zeng et al., 2025).

Roxadustat has the potential to stabilize HIF-1α, thereby simulating hypoxic signals. The activation of HIF-1α encourages a metabolic shift towards aerobic glycolysis, allowing tumor cells to proliferate under hypoxic conditions. Empirical studies have demonstrated that Roxadustat markedly upregulates the expression of glucose transporters, including GLUT1, and glycolytic enzymes, such as LDHA and PDK1, through the activation of the HIF signaling pathway. This activation enhances cellular glycolytic metabolism and optimizes energy supply (Makinen et al., 2024; Pullamsetti et al., 2020).

Under hypoxic conditions, a metabolic transition occurs from oxidative metabolism to reductive carboxylation, mediated by a mechanism involving HIF-1. In hypoxic glioblastoma cells, the predominant source of citrate is glutamine, processed through reductive carboxylation. Importantly, this cell line exhibits an inability to proliferate when deprived of citrate (Infantino et al., 2021). Dysregulation of lipid metabolism, particularly in fatty acid synthesis and catabolism, is a prevalent characteristic of cancer cells. HIF-1α plays a regulatory role in enzymes associated with lipid biosynthesis, which is crucial for membrane formation and energy production. This metabolic reprogramming provides tumor cells with a survival advantage, allowing them to adapt to various microenvironmental conditions. Consequently, the optimization of lipid metabolic pathways constitutes a critical mechanism underlying the adaptive capacity of cancer cells (Mylonis et al., 2019). HIF-1α is known to upregulate the expression of fatty acid-binding proteins (FABPs), which are essential for the transport of fatty acids. In human glioblastoma cells, research by Bensaad et al. has demonstrated that HIF-1α is crucial for the induction of FABP3 and FABP7, subsequently facilitating lipid droplet accumulation (Jun et al., 2017).

The pentose phosphate pathway is integral to the biosynthesis of NADPH and essential metabolites such as ribose-5-phosphate, which are vital for various anabolic processes within the body. The activation of HIF-1α increases the flux through this pathway, thereby enhancing the biosynthesis of crucial molecules and boosting antioxidant activity. This metabolic pathway is particularly well-adapted to satisfy the specific metabolic requirements of highly proliferative cancer cells (Stincone et al., 2015). Beyond reshaping energy metabolism, the metabolic shift induced by HIF-1α also creates a hypoxic and nutrient-deficient milieu that necessitates vascular remodeling. This forms the biological bridge to one of HIF-1α′s most studied effects—angiogenesis.

## Angiogenesis: microenvironment remodeling

Angiogenesis is integral to tumor growth and metastasis, as the formation of new blood vessels supplies tumors with essential oxygen, nutrients, and pathways for metastatic spread. HIF-1α can bind to hypoxia response elements (HREs) on the promoter region of the VEGF gene, thereby directly enhancing the transcriptional expression of VEGF. As one of the most potent angiogenic factors, VEGF facilitates the proliferation, migration, and survival of endothelial cells (Basheeruddin and Qausain, 2024).

Empirical studies have shown that Roxadustat can enhance the angiogenic activity of human umbilical vein endothelial cells (HUVECs) while concurrently upregulating the HIF-1α/VEGF/VEGFR2 signaling pathway. This mechanism may result in the formation of new blood vessels surrounding tumors, thereby supplying additional nutrients and oxygen and ultimately promoting tumor growth (Zhu et al., 2019).

*In vivo* studies conducted on mice have demonstrated that Roxadustat induces an upregulation of HIF-1α and VEGF expression levels, exhibiting a clear dose-dependent relationship (Lan et al., 2023). Conversely, research utilizing a spontaneous breast cancer model characterized by high VEGF sensitivity revealed that treatment with FG-4497, a HIF-PHI drug of the same class as Roxadustat, did not enhance tumorigenesis or progression, even though VEGF levels were elevated (Seeley et al., 2017). While angiogenesis provides tumors with the necessary oxygen and nutrients, it also facilitates dissemination. The same HIF-1α–VEGF axis that drives vessel formation can enhance tumor invasiveness through epithelial–mesenchymal transition (EMT) and extracellular matrix degradation.

## Apoptosis resistance: enhanced survival signal

HIF-1α enhances the expression of anti-apoptotic proteins, thereby inhibiting programmed cell death and promoting cellular survival. B-cell lymphoma 2 (Bcl-2), a principal anti-apoptotic protein, impedes apoptosis by preventing mitochondrial outer membrane permeabilization and is involved in cell cycle regulation (Infantino et al., 2021).

HIF-1α modulates apoptosis through the transcriptional regulation of pro-apoptotic and anti-apoptotic proteins such as BNIP-3, BNIP-3L, BCL-2, BCL-XL, BAX, and NOXA, or through the stabilization of p53 (Chen et al., 2003). Studies have demonstrated that under hypoxic conditions, HIF-1α can stabilize wild-type p53. For example, in A204 rhabdomyosarcoma (RMS) cells, which possess wild-type p53, the stability of the p53 protein is enhanced under hypoxia, a process associated with the activation of HIF-1α (Kilic et al., 2007). Another study demonstrated that in non-small cell lung cancer (NSCLC), mutant p53 interacts with HIF-1α to form a complex, thereby enhancing the transcriptional activity of HIF-1α on hypoxia-responsive genes and promoting tumor progression. In contrast, under normoxic conditions, wild-type p53 negatively regulates the stability of HIF-1α by binding to its DNA-binding domain, inhibiting its transcriptional activity and consequently suppressing the hypoxic adaptation and survival of tumor cells (Amelio et al., 2018).

HIF-1α can also inhibit cell apoptosis through other mechanisms, such as upregulating GLUT-1 expression to enhance glucose uptake and glycolysis. For example, in pancreatic cancer, constitutively expressed HIF-1α activates anaerobic metabolic pathways, enabling pancreatic cancer cells to survive and proliferate in hypoxic and nutrient-deprived environments, thereby increasing their resistance to apoptosis (Akakura et al., 2001).

## Immune escape and chemoresistance

Tumor immune evasion is a critical factor in cancer pathogenesis and malignant progression, primarily facilitated by the interaction between programmed cell death ligand 1 (PD-L1) on tumor cells and the PD-1 receptor on immune cells. The binding of PD-L1 to PD-1 initiates signaling pathways that enable tumor cells to escape immune surveillance, thereby promoting tumor growth and metastasis. Recent research has shown that HIF-1α can directly associate with the HRE-4 within the PD-L1 promoter region, leading to a rapid and substantial upregulation of PD-L1 expression on the surface of tumor cells under hypoxic conditions. This upregulation of PD-L1 facilitates its binding to the PD-1 receptor on T cells, inducing T cell apoptosis and consequently allowing tumor cells to evade the host immune response (Chen et al., 2015).

Under hypoxic conditions, the upregulation of HIF-1α can enhance the suppressive function of myeloid-derived suppressor cells (MDSCs) by directly promoting the transcription of PD-L1 within these cells. This, in turn, leads to the inhibition of T cell activation due to increased PD-L1 expression. Through the application of chromatin immunoprecipitation and luciferase reporter assays, Muhammad and colleagues elucidated that HIF-1α binds to the transcriptionally active hypoxia-response element within the PD-L1 promoter. Moreover, *ex vivo* inhibition of PD-L1 in MDSCs, achieved through the use of an anti-PD-L1 monoclonal antibody, resulted in a significant reduction in the expression of protumor cytokines, including interleukin-6 (IL-6) and interleukin-10 (IL-10) (Wang et al., 2025).

The overexpression of the drug efflux pump multidrug resistance 1 (MDR1) gene and its encoded P-glycoprotein is significantly implicated in the development of multidrug resistance. Another critical drug transporter contributing to drug resistance is the multidrug resistance-associated protein 1 (MRP1). HIF-1α upregulates both MDR1 and MRP1 by directly binding to HRE sites within their gene promoter regions (Song et al., 2025).

Given its central role in modulating the cellular response to hypoxia, HIF-1α has emerged as a crucial target in cancer therapy. Recent advancements in cancer research have concentrated on developing therapeutic strategies targeting HIF-1 pathways. These strategies include the utilization of small molecules derived from natural products, which possess unique chemical structures and biological activities capable of inhibiting HIF-1 signaling. Certain HIF-1 inhibitors, such as PX-478, PMX290, and FK228, have demonstrated the ability to enhance the antitumor effects of chemotherapy and radiotherapy by inducing apoptosis in pancreatic ductal adenocarcinoma and various other cancers (Bui et al., 2022).

**FIGURE 1:**
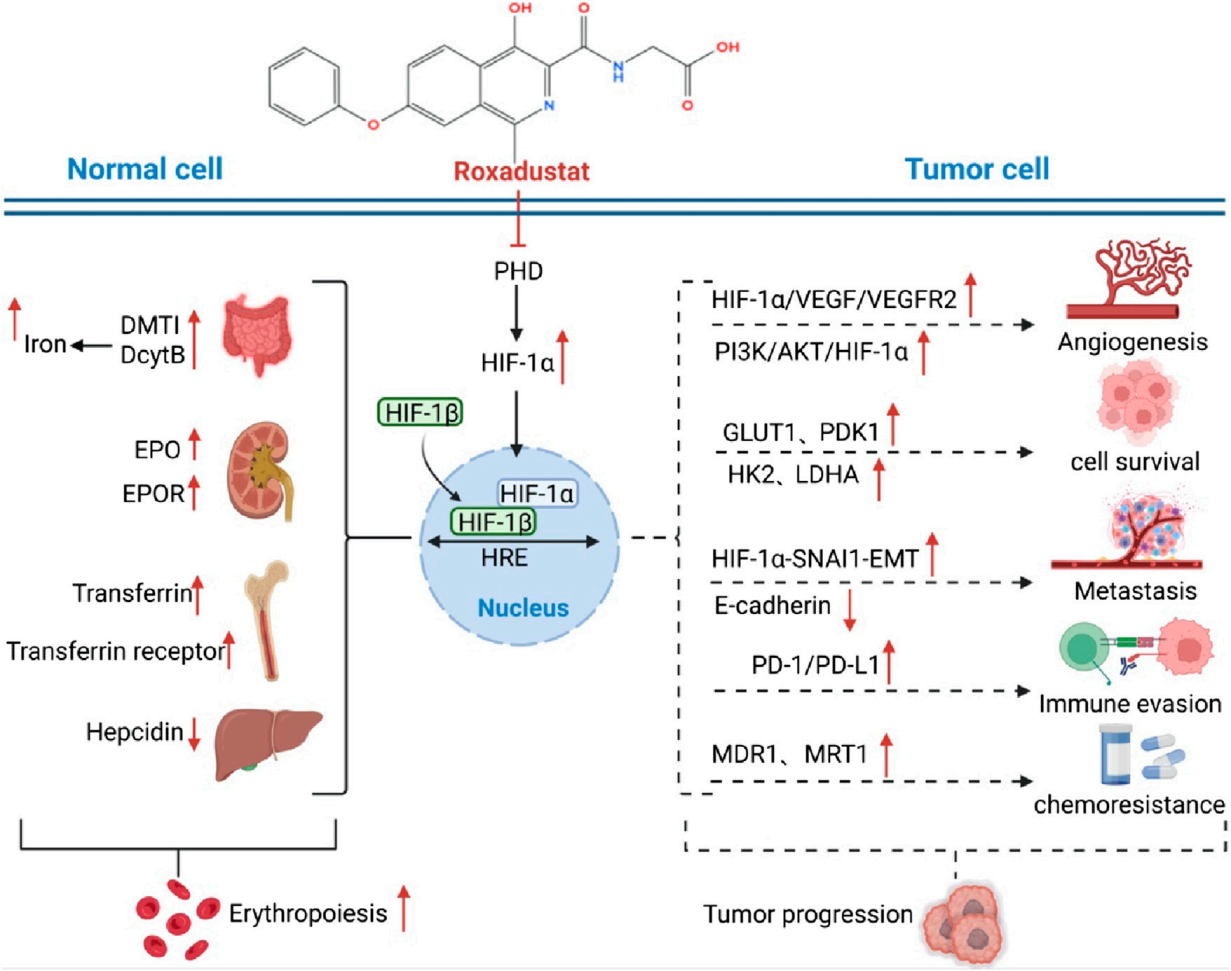
Dual mechanisms of Roxadustat in anemia improvement and potential tumor progression. **Normal Erythropoiesis:** Roxadustat inhibits PHD, stabilizes HIF-1α, and promotes EPO and EPOR expression. This enhances downstream signaling, stimulating bone marrow hematopoietic stem cell proliferation and differentiation, thereby generating red blood cells and improving anemia. It also upregulates DMT1 and DcytB to increase iron uptake and utilization, supporting erythropoiesis. **Tumor Cell Impact:** In tumor cells, Roxadustat’s inhibition of PHD stabilizes HIF-1α, which binds to HIF-1β to form the active HIF-1 complex. This activates the PI3K/AKT/HIF-1α pathway, increasing the expression of GLUT1, HK2, and LDHA, thus promoting glucose uptake and metabolism to provide energy for tumor growth. HIF-1α also interacts with SNAI1 to induce EMT, reducing E-cadherin expression and enhancing tumor invasion and metastasis. Additionally, Roxadustat upregulates PD-1/PD-L1 expression on tumor cells, enabling immune evasion. Furthermore, it may regulate MDR1 and MRT1, contributing to chemoresistance.

As previously discussed, mounting evidence indicates that HIF-1α facilitates tumor cell proliferation through various mechanisms. Figure 1 presents a comprehensive overview of the dual mechanisms of Roxadustat, highlighting its potential to enhance tumor growth via specific pathways and genes, alongside its role in promoting erythropoiesis. Furthermore, therapeutic agents aimed at inhibiting HIF-1α signaling have been investigated for their efficacy in cancer treatment. In this regard, the potential risk of exacerbating tumor growth through the activation of HIF-1α signaling in the management of cancer-related anemia necessitates thorough evaluation. Given the multiple pro-tumor mechanisms of HIF-1α activation, it becomes essential to determine whether these preclinical concerns translate into clinical risk. The following section therefore reviews available clinical evidence, bridging mechanistic insights from bench to bedside, and evaluating whether HIF-1α stabilization by Roxadustat indeed correlates with tumor progression in cancer patients.

## Clinical evidence on the safety of tumor progression in CIA treated with Roxadustat

In 2019, the U.S. Food and Drug Administration (FDA) approved Roxadustat for the treatment of renal anemia in individuals with chronic kidney disease (CKD). Long-term follow-up studies involving CKD patients have not demonstrated a significant increase in the incidence of malignant tumors associated with Roxadustat use (Du et al., 2025). However, the potential for Roxadustat to promote tumor progression or induce drug resistance in patients with pre-existing tumors remains an area necessitating further investigation. In a Japanese real-world study assessing the safety and efficacy of Roxadustat, “malignant tumors” were identified as a category of adverse reactions warranting special concern within the safety outcomes. Although a definitive causal link between Roxadustat and tumor progression has not been conclusively established, the data indicated that 12 patients (0.6%) from the overall study population experienced malignant tumor-related adverse drug reactions (ADRs). These reactions encompassed 13 distinct tumor types, including lung cancer, prostate cancer, and acute myeloid leukemia. Notably, the study provided detailed information regarding the tumor history of these patients: among the 13 cases of malignant tumors, 7 patients had a prior history of tumors (such as gastric cancer and colorectal cancer), while 6 patients had no relevant history. This suggests that some cases may represent newly developed tumors, whereas others may be associated with a pre-existing medical history (Tsuruya et al., 2025).

In an open-label Phase II study, patients with non-myeloid malignancies and CIA were enrolled and received oral Roxadustat treatment for a maximum duration of 16 weeks. Data on TESAEs showed that 7.6% of patients died due to cancer metastasis, including metastatic pancreatic cancer, cholangiocarcinoma, ovarian cancer, and metastatic small cell lung cancer (SCLC) (Glaspy et al., 2023). Similarly, in Phase III clinical trial evaluating the efficacy and safety of Roxadustat in the treatment of CIA in patients with non-myeloid malignancies, its supplementary tables recorded TEAEs that led to death (Lu et al., 2025). In the cohort receiving Roxadustat, nine patients succumbed to TEAEs, with four cases attributed to tumor progression, including instances of lung metastasis and malignant tumor advancement. While these events were deemed unrelated to the investigational drug, it is important to note that the study’s observation period was relatively brief, spanning from several months to 1 year from patient enrollment to the final follow-up. This limited timeframe may not be sufficient for a comprehensive assessment of tumor progression. The summary of studies on Roxadustat and related research is shown in Table 1.

**TABLE 1:** Summary of key preclinical and clinical studies evaluating Roxadustat or related HIF-PHIs and their oncologic implications.

Study	Year	Model/Study design	Population/Experimental system	Intervention (HIF-PHI)	Key findings	Relevance to HIF-1α/Oncologic risk
Li et al. (2025)	2025	Phase III, randomized, open-label, active-controlled	Non-myeloid malignancy with CIA	Roxadustat vs. ESA	Roxadustat non-inferior to ESA for Hb improvement; TEAEs comparable	No tumor-progression signal; follow-up <1 year
Glaspy et al. (2023)	2023	Phase II, open-label	Solid-tumor patients receiving multi-cycle chemotherapy	Roxadustat	Effective anemia correction; 7.6% deaths due to cancer metastasis	No causal link established; highlights need for longer surveillance
Tsuruya et al. (2025)	2025	Real-world, post-marketing surveillance	CKD patients (Japan)	Roxadustat	0.6% ADRs related to malignant tumors; mostly with prior cancer history	No signal for new malignancy; confounding by prior tumors possible
Du et al. (2025)	2025	Long-term registry (ROXSTAR)	CKD anemia	Roxadustat	Sustained Hb correction; no significant rise in malignancy	Suggests renal safety; oncology-specific risk untested
Zhu et al. (2019)	2019	*In vivo* rat wound-healing model	Diabetic rats	Roxadustat	Promotes angiogenesis via HIF-1α/VEGF/VEGFR2 signaling	Confirms pathway activation; potential tumor relevance
Lan et al. (2023)	2023	*In vivo* rat skin-flap model	Normal rats	Roxadustat	Upregulated HIF-1α and VEGF; enhanced tissue survival	Dose-dependent HIF-1α activation; theoretical oncogenic risk
Seeley et al. (2017)	2017	VEGF-sensitive breast-cancer model	MMTV-PyMT mice	FG-4497 (class analog)	Did not promote tumor initiation or metastasis	Suggests not all HIF-PHIs enhance tumor growth
Bui et al. (2022)	2022	Review/mechanistic analysis	Multiple tumor models	HIF-1 inhibitors	HIF-1 blockade enhances chemo-radiotherapy efficacy	Highlights opposite, therapeutic modulation of HIF-1α

Abbreviations: CIA, chemotherapy-induced anemia; ESA, erythropoiesis-stimulating agent; CKD, chronic kidney disease; ADR, adverse drug reaction; TEAE, treatment-emergent adverse event; HIF-PHI, hypoxia-inducible factor prolyl hydroxylase inhibitor.

## Conclusion

In summary, Roxadustat effectively treats renal anemia by stimulating erythropoietin production and regulating iron metabolism. However, its activation of HIF-1α in the tumor microenvironment may promote tumor progression through mechanisms such as angiogenesis, glycolysis enhancement, and immune evasion. Current clinical data offer short-term reassurance but are limited by sample size and duration. Bridging the gap between theoretical oncogenic risk and clinical safety requires well-powered, long-term, tumor-specific studies. Until such evidence is available, Roxadustat should be used with prudence in oncology, balancing hematologic benefit against potential tumor-related uncertainty.
